# Retinal metastasis from lung adenocarcinoma: a case report

**DOI:** 10.3389/fonc.2025.1616331

**Published:** 2025-08-04

**Authors:** Danfeng Li, Lifu Luo, Bo Yang, Jun Xiao

**Affiliations:** Department of Ophthalmology, The Second Hospital of Jilin University, Changchun, Jilin, China

**Keywords:** retinal metastasis, lung adenocarcinoma, ocular metastasis, multimodal imaging, case report, optical coherence tomography

## Abstract

Retinal metastasis is an exceedingly rare manifestation of systemic cancer, accounting for less than 1% of ocular metastases. Lung adenocarcinoma is among the primary tumors that can metastasize to the retina, though such cases are seldom reported. We present a 70-year-old male with a history of lung adenocarcinoma who presented with a decline in vision in his left eye. Fundus examination revealed a large yellow-white lesion with hemorrhage in the macula and temporal retina. Multimodal imaging, including optical coherence tomography (OCT) and fluorescein angiography (FA), confirmed the diagnosis of retinal metastasis. This case underscores the importance of considering retinal metastasis in cancer patients with visual disturbances. Early diagnosis through comprehensive ophthalmic evaluation is crucial to avoid misdiagnosis.

## Introduction

Tumor metastasis to the eye is a rare but significant complication of systemic cancer, with the choroid being the most commonly affected site, accounting for approximately 90% of cases. In contrast, retinal metastasis is exceedingly rare, comprising less than 1% of ocular metastases (1). The retina’s unique anatomical and physiological characteristics, including its dual blood supply and the presence of the blood-retinal barrier, may contribute to the low incidence of retinal metastasis. However, when it occurs, it is often associated with advanced systemic disease and carries a poor prognosis.

Typically, it presents as a solitary lesion in one eye, appearing as irregular yellow-white patches on the retina. These lesions closely resemble necrotizing retinitis caused by conditions such as ocular toxoplasmosis or viral infections. In this case, we present an elderly male patient diagnosed with lung adenocarcinoma who sought medical care due to a decline in vision. Following a detailed review of his medical history and evaluation through fundus multimodal imaging, the condition was identified as retinal metastasis.

## Case presentation

**Figure 1:**
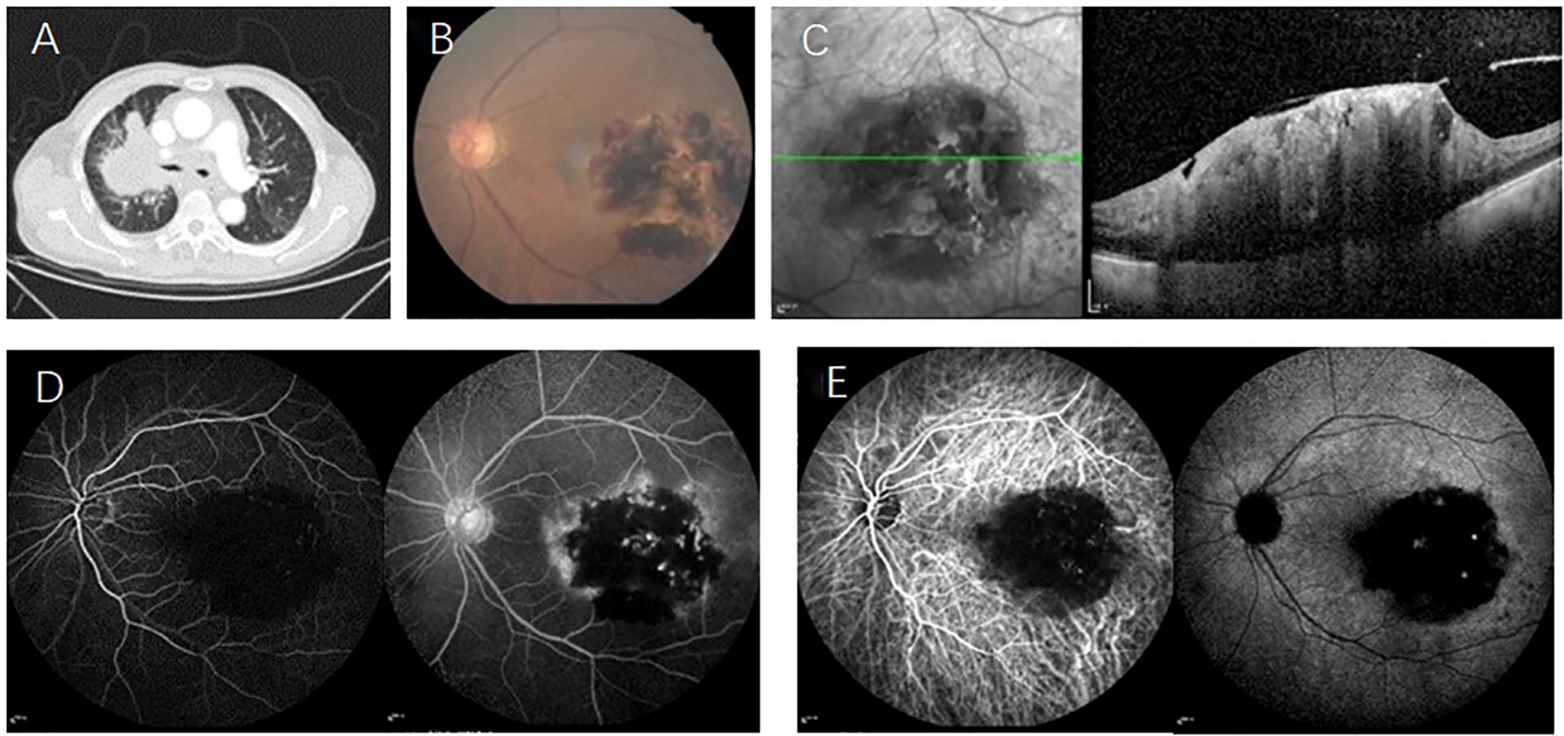
**(A)** CT scan of the lungs reveals a central-type lung cancer located in the upper lobe of the right lung; **(B)** Fundus examination identifies large yellow-white lesions accompanied by bleeding in the macula and the temporal retina of the left eye; **(C)** OCT demonstrates retinal protrusion and necrotic changes affecting the entire retina; **(D)** FA highlights an extensive area of low fluorescence in the posterior pole of the retina, with multiple punctate areas of high fluorescence within the tumor and evidence of late-stage leakage; **(E)** ICGA shows persistent low fluorescence in the posterior pole of the retina, with visibly dilated retinal blood vessels within the affected area.

A 70-year-old male presented to our hospital complaining of decrease of vision in his left eye for one months. Four months earlier, he had been diagnosed with lung adenocarcinoma accompanied by cervical lymph node metastasis (**Figure 1A**) and opted for traditional Chinese medicine treatment rather than systemic anti-tumor therapy. At the time of presentation, the best-corrected visual acuity for the left eye was recorded as counting fingers, Due to a history of ischemic optic neuropathy, the patient now has optic nerve atrophy with hand motion vision. Fundus photography of the left eye revealed a large yellow-white lesion with associated hemorrhage located at the macula and temporal retina (**Figure 1B**). Optical coherence tomography (OCT) demonstrated retinal protrusion and necrotic changes involving the entire retinal layer (**Figure 1C**). Fluorescein angiography (FA) highlighted a large region of hypofluorescence at the posterior pole of the retina, interspersed with multiple hyperfluorescent dots within the lesion and late-stage leakage (**Figure 1D**). Indocyanine green angiography (ICGA) showed persistent low fluorescence at the posterior pole of the retina, with visibly dilated retinal blood vessels within the lesion (**Figure 1E**).

**Figure 2:**
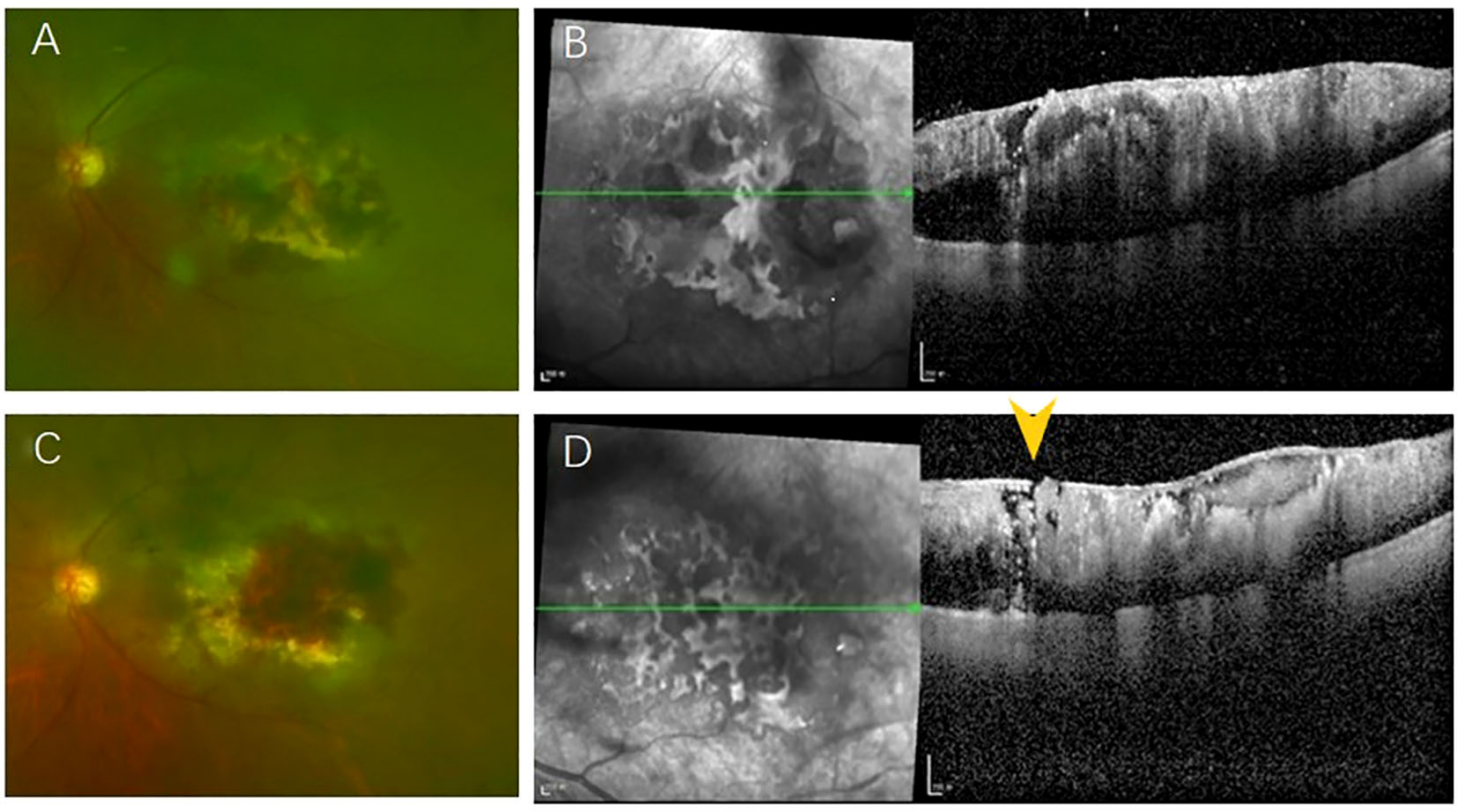
**(A)**The lesion in the macular area has expanded compared to the previous observation, while the bleeding at the lesion site has decreased; **(B)** OCT imaging indicates that the tumor area has further enlarged, yet the degree of retinal protrusion has diminished; **(C)** The fundus lesion has undergone significant enlargement, accompanied by extensive areas of bleeding over the mass; **(D)** OCT reveals a rupture in the internal limiting membrane (yellow arrow) and a collapse of the tumor lesion.

After 20 days, the lesion in the macular area showed expansion compared to the initial observation, while bleeding at the lesion site decreased (**Figure 2A**). OCT imaging revealed that the tumor area had further enlarged, though the degree of retinal protrusion was reduced (**Figure 2B**). At the one-month follow-up, the patient’s fundus lesions exhibited significant progression, with extensive hemorrhage over the mass and increased vitreous opacity compared to previous findings (**Figure 2C**). OCT scans indicated internal limiting membrane rupture and collapse of the tumor lesion (**Figure 2D**). Given the diagnosis of retinal metastasis linked to lung cancer with widespread multi-organ metastasis, the patient opted for observation.

## Discussion

Tumor metastasis refers to the spread and growth of tumor cells in tissues and organs located far from their primary site. The main modes of metastasis include hematogenous or lymphatic dissemination, direct infiltration into adjacent tissues, and implantation in distant organs (2). Tumors can metastasize to ocular tissues via the bloodstream, with the choroid being the most common site, accounting for 90% of cases. The most frequently affected ocular structures are the choroid, iris, and ciliary body, in that order (1). Retinal metastasis is rare, possibly due to the presence of internal and external barriers that limit tumor cells from infiltrating the retina, resulting in fewer than 1% of ocular metastasis cases. When retinal involvement occurs, it typically begins in the retinal nerve fiber layer and ganglion cell layer. The superficial capillary plexus, supplied by the central retinal artery, serves as the primary pathway for tumor cell metastasis. As the retinal tumor progresses, it invades deeper retinal layers. Once full-thickness retinal involvement occurs, tumor cells may penetrate the retinal pigment epithelium/Bruch’s membrane to reach the choroid. However, cases of choroidal involvement are rare, perhaps because the existence of tight junction of the RPE prevents the spread of cancer cell (3). In addition, cancer cells may also penetrate the internal limiting membrane and spread into the vitreous cavity (4). In the final OCT imaging during this patient’s follow-up, findings revealed internal limiting membrane rupture, retinal tissue collapse, and worsening vitreous opacity, all of which are indicative of vitreous metastasis.

Among all cases of retinal metastasis, cutaneous melanoma is the most common primary tumor. A study (5) analyzing 69 patients with retinal metastatic cancer identified skin melanoma (36%), lung cancer (23%), gastrointestinal cancers (17%), and breast cancer (12%) as the primary sources. For some patients, retinal metastasis may be the initial manifestation of the disease, with the primary tumor site remaining unknown. In such cases, a thorough search for the primary lesion and systemic metastasis evaluation is essential.

Retinal metastatic carcinoma is generally unilateral and localized, though reports of bilateral involvement and multifocal lesions also exist (6). Clinical symptoms of retinal metastasis include decreased vision, floaters, eye pain, and other issues (7). Fundoscopic examination typically reveals irregular yellow-white retinal lesions. Clinical presentations included patchy retinal infiltrate, elevated retinal mass, and punctate retinal infiltrate, which closely resemble the features of necrotizing retinitis secondary to ocular toxoplasmosis or viral infections (8). Retinal metastases originating from cutaneous melanoma may appear brown and often exhibit accompanying vitreous dissemination (9). Other possible manifestations include retinal vascular sheathing, retinal hemorrhage, vitreous opacity, exudative retinal detachment, and in the advanced stages, secondary glaucoma due to tumor cell obstruction of the anterior chamber angle (1). Given the rarity of retinal metastasis, misdiagnosis as infectious retinitis is common. Fluorescein angiography and indocyanine green angiography typically reveal early hypofluorescence, followed by vascular dilation in the affected area. On OCT, retinal metastatic lesions present as highly reflective masses within the inner or full retina, often accompanied by subretinal fluid, intraretinal fluid, and hyperreflective substances between retinal layers (5).

Currently, treatment options for retinal metastasis remain limited due to insufficient data. Management strategies include observation, intravitreal injection of chemotherapeutic agents, and localized radiotherapy. In some cases, enucleation of the affected eye may be necessary (6). Additionally, reports have indicated that systemic chemotherapy plays a positive role in the regression of retinal metastatic lesions (10). When a tumor metastasizes to the retina, it is generally indicative of terminal-stage disease, with a poor prognosis. The median survival time from diagnosis of retinal metastasis to death reported in the literature ranges from 5.7 to 11 months (3, 4). In this case, systemic anti-tumor treatment was not pursued due to the patient’s advanced lung cancer with extensive metastatic involvement, including the eyes and lymph nodes.

## Conclusion

This case report illustrates the diagnostic challenges and clinical significance of retinal metastasis in a patient with lung adenocarcinoma. Retinal metastasis, though rare, should be considered in cancer patients presenting with visual disturbances, particularly those with advanced systemic disease. Multimodal imaging plays a crucial role in confirming the diagnosis and guiding management. Despite the poor prognosis associated with retinal metastasis, early recognition and intervention can help preserve vision and improve patient outcomes.

## Data Availability

The original contributions presented in the study are included in the article/supplementary material. Further inquiries can be directed to the corresponding author.
